# Rotundifuran Induces Ferroptotic Cell Death and Mitochondria Permeability Transition in Lung Cancer Cells

**DOI:** 10.3390/biomedicines12030576

**Published:** 2024-03-05

**Authors:** Myung-Ji Kang, Dong-Oh Moon, Ji-Yoon Park, Namho Kim, Su Hyeon Lee, Hyung Won Ryu, Yang Hoon Huh, Hyun-Sun Lee, Mun-Ock Kim

**Affiliations:** 1 Natural Product Research Center, Korea Research Institute of Bioscience and Biotechnology (KRIBB), Cheongju 28116, Republic of Korea; kmj4363@naver.com (M.-J.K.); parkgyun98@kribb.re.kr (J.-Y.P.); skagh6684@kribb.re.kr (N.K.); hyeon99@kribb.re.kr (S.H.L.); ryuhw@kribb.re.kr (H.W.R.); leehs@kribb.re.kr (H.-S.L.); 2 Department of Biology Education, Daegu University, 201, Daegudae-ro, Gyeongsan-si 38453, Republic of Korea; domoon@daegu.ac.kr; 3 Department of Anatomy & Cell Biology, Department of Medical Science, College of Medicine, Chungnam National University, Daejeon 35015, Republic of Korea; 4 College of Pharmacy, Chungbuk National University, Cheongju 28160, Republic of Korea; 5 Electron Microscopy Research Center, Korea Basic Science Institute (KBSI), Cheongju 28119, Republic of Korea; hyh1127@kbsi.re.kr

**Keywords:** rotundifuran, ferroptosis, reactive oxygen species (ROS), mitochondria permeability transition (MPT)

## Abstract

Rotundifuran (RF), a potent anti-inflammatory and anti-cancer compound, is a natural compound predominantly present in *Vitex Rotundifolia*. Herein, we investigated the effects of RF on the growth of lung cancer cells. Our findings suggested that RF inhibits cell growth, highlighting its potential as a therapeutic agent for cancer treatment. Interestingly, we observed that cell growth inhibition was not due to apoptosis, as caspases were not activated and DNA fragmentation did not occur. Furthermore, we found that intracellular vacuoles and autophagy were induced, but RF-induced cell death was not affected when autophagy was inhibited. This prompted us to investigate other possible mechanisms underlying cell growth inhibition. Through a cDNA chip analysis, we confirmed changes in the expression of ferroptosis-related genes and observed lipid peroxidation. We further examined the effect of ferroptosis inhibitors and found that they alleviated cell growth inhibition induced by RF. We also observed the involvement of calcium signaling, ROS accumulation, and JNK signaling in the induction of ferroptosis. Our findings suggested that RF is a potent anti-cancer drug and further studies are needed to validate its clinal use.

## 1. Introduction

Lung cancer is a common and fatal cancer that affects many people worldwide. Although diagnostic and treatment methods have been steadily developed, the 5-year survival rate of patients with lung cancer is very low [1,2,3,4]. Therefore, strategies for developing effective treatments for lung cancer are necessitated.

Recently, ferroptosis, a nonapoptotic form of programmed cell death characterized by iron-dependent lipid peroxidation, has emerged as a potential therapeutic strategy for various cancers, including lung cancer. Ferroptosis is induced by the accumulation of lipid peroxides generated by an imbalance between the production of reactive oxygen species (ROS) and the cellular antioxidant defense system in cells [5,6,7,8]. Ferroptosis is distinct from other forms of cell death, including apoptosis, necroptosis, and autophagy, in terms of morphology, biochemical mechanisms, and genetic requirements.

In lung cancer, ferroptosis is a promising approach for suppressing cancer growth and enhancing the effectiveness of chemotherapy and radiation therapy [9,10]. Research has indicated that lung cancer cells are particularly susceptible to ferroptosis owing to their elevated metabolic rate, rapid proliferation, and increased iron uptake [11,12]. Furthermore, several molecular pathways that regulate ferroptosis are dysregulated in lung cancer, rendering the cancer cells more sensitive to ferroptosis. These pathways include the tumor suppressor protein p53, the lipid peroxidation enzyme 15-lipoxygenase (15-LOX), and the iron metabolism regulator transferrin receptor 1 (TFRC) [13,14].

One of the main challenges in developing ferroptosis-based therapies for lung cancer is identifying the most effective ferroptosis inducers and understanding the molecular mechanisms of their action. Several compounds have been shown to induce ferroptosis in lung cancer cells, including erastin, RSL3, and FIN56. These compounds work by targeting various components of the ferroptosis pathway, including the glutathione peroxidase 4 (GPX4), the cystine/glutamate antiporter system xc^-^, and the iron metabolism regulator system [15,16].

The role of ferroptosis in lung cancer is supported by preclinical studies demonstrating that ferroptosis induction enhances the sensitivity of lung cancer cells to chemotherapy and radiation therapy. A recent study demonstrated that the combination of erastin and cisplatin, a commonly used chemotherapeutic drug, resulted in a substantial reduction in tumor growth and prolonged survival in a mouse model of lung cancer [17]. Similarly, another study reported that the combination of RSL3 and ionizing radiation-induced ferroptosis in lung cancer cells significantly increased tumor cell death [18]. Recent studies have focused on identifying the mechanisms regulating the sensitivity of lung cancer cells to ferroptosis. Several studies have reported that genetic mutations or the dysregulation of specific signaling pathways in lung cancer cells can affect their sensitivity to ferroptosis induction. For example, loss-of-function mutations in GPX4 or the system xc^-^ antiporter have been shown to sensitize lung cancer cells to ferroptosis induction [19]. Furthermore, the activation of the NF–κB pathway, a pivotal regulator of inflammation and immune responses, hinders the induction of ferroptosis in lung cancer cells [20,21].

Rotundifuran (RF) is a natural compound found in many medicinal plants, including traditional Chinese medicine [22,23,24,25]. It has been shown to have potent anti-inflammatory and anti-tumor effects, making it a promising candidate for cancer therapy. However, despite its potential, relatively little research has been conducted on the use of RF in lung cancer treatment. Investigating the potential of RF to effectively induce ferroptosis, leading to cell death in lung cancer cell lines, along with research on the signal transduction pathways involved in this process, is essential for obtaining critical data as a basic, preclinical study.

## 2. Materials and Methods

### 2.1. Reagents and Materials

Chloroquine (CQ), N-acetyl-L-cysteine (NAC), 3-(4,5-dimethyl-2-thiazolyl)-2,5-diphenyl-2H-tetrazolium bromide (MTT), carbonyl cyanide m-chlorophenylhydrazone (CCCP), and liproxstatin-1 were sourced from Sigma-Aldrich (St. Louis, MO, USA). The caspase-8, caspase-9, and caspase-3 colorimetric assay kits, along with z-VAD-fmk (a pan-caspase inhibitor), were acquired from R&D Systems (Minneapolis, MA, USA). Specific antibodies against phosphorylated (p)-JNK and ATF3 were purchased from Santa Cruz Biotechnology (Dallas, TX, USA). Anti-tubulin, LC3, Beclin1 ATG5, GPX4, SAT1, and SQSTM1/p62 antibodies were purchased from Cell Signaling Technology (Beverly, MA, USA). Anti-4HNE, Rhod-2, and Ruthenium Red antibodies were purchased from Abcam (Cambridge, UK). Additionally, Fluo4-AM, MitoSOX, and BAPTA were purchased from Invitrogen (Carlsbad, CA, USA). RF was provided by Dr. Hyun Sun Lee (Korea Research Institute of Bioscience and Biotechnology, Cheongju, Republic of Korea).

### 2.2. Cell Culture

A549, MCF-7, HepG2, H292, SW480, and U937 cell lines were obtained from the American Type Culture Collection (ATCC, Manassas, VA, USA). A549, MCF-7, and U937 cells were maintained in an RPMI medium, whereas HepG2, H292, and SW480 cells were grown in a DMEM medium (Welgene, Gyeongsan-si, Republic of Korea). The media were supplemented with 10% fetal bovine serum (Gibco, Rockville, MD, USA) and 1% penicillin–streptomycin (Gibco, Rockville, MD, USA). The cultures were maintained at 37 °C in a 5% CO_2_ atmosphere and 100% humidity.

### 2.3. Cell Viability

The MTT assay relies on the conversion of MTT to formazan crystals, catalyzed by mitochondrial enzymes within viable cells. Subsequently, formazan crystals were quantified using a spectrophotometer.

### 2.4. Caspase Enzyme Activity

Commercially available total colorimetric assay kits for caspase-8, -9, and -3 were used to evaluate their activities in A549 cells exposed to RF. Duplicate samples were tested following the guidelines provided by the manufacturer.

### 2.5. Cytosolic and Mitochondrial Ca^2+^ Levels

A549 cells were treated with either Fluo-4 AM or Rhod-2 AM at a concentration of 1 µg/mL, followed by incubation at 37 °C for 15 min. Subsequently, the samples were promptly analyzed using a flow cytometer (CytoFLEX; Beckman Coulter, Brea, CA, USA).

### 2.6. Transmission Electron Microscopy

A total of 1 × 10^5^ A549 cells/mL was cultured in 6-well plates for 1 and 6 h and, subsequently, treated with 40 µM RF. Cells were collected, fixed with 2.5% glutaraldehyde and 1% osmium tetroxide on ice for 2 h, and washed with phosphate-buffered saline (PBS). The tissues were then dehydrated in ethanol and propylene oxide in series, embedded in an Epon 812 mixture, and polymerized in an oven at 70 °C for 24 h. The sections acquired from the polymerized blocks were collected on grids, counterstained with uranyl acetate and lead citrate, and examined using a JEM-1400Plus at 120 kV (JEOL, Tokyo, Japan).

### 2.7. Transfection of siRNA

A549 cells were inoculated in 6-well plates at a density of 16 × 10^5^/well, and the Lipofectamine 2000 reagent was used to transfect ATG5 siRNA or non-targeted siRNA (Thermo Fisher Scientific, Waltham, MA, USA) at a final concentration of 200 nM. After transfection for 48 h, the cells were detected using Western blotting.

### 2.8. Intracellular ROS Measurement

The levels of reactive oxygen species (ROS) were assessed utilizing MitoSOX (mitochondrial superoxide indicator) probes. Cells were seeded at a density of approximately 5 × 10^4^ cells/mL in a 24-well plate and treated with RF. Following trypsinization, cells were incubated with 1 μM MitoSOX for 20 min in the dark at 37 °C. Fluorescence was quantified using a flow cytometer (CytoFLEX; Beckman Coulter, Brea, CA, USA).

### 2.9. Cell Cycle Analysis

After the specified treatment, A549 cells were stained with PI dye (1 mg/mL) in the dark at 37 °C for 20 min. Subsequently, the cells were washed with PBS and analyzed using a flow cytometer (CytoFLEX; Beckman Coulter, Brea, CA, USA).

### 2.10. RHOD-2 Staining

The A549 cells were exposed to RF for 24 h. Subsequently, the cells were incubated with pre-warmed Hoechst for 20 min at 37 °C in the dark. After another PBS wash, the cells were visualized under a fluorescence microscope (ZEISS, Jena, Germany).

### 2.11. Lipid Peroxidation and Intracellular Ferrous Levels

To assess lipid peroxidation, we used a C11-BODIPY 581/591 probe. A549 cells were treated with C11-BODIPY 581/591 (1 µM) for 30 min. FerroOrange was used to measure intracellular ferrous ions. The fluorescence signals in the green (484/510 nm) and red (581/610 nm) channels were observed using a fluorescence microscope (ZEISS, Jena, Germany).

### 2.12. Mitochondrial Membrane Potential

After the specified treatment, A549 cells were treated with DiOC6(3) dye (40 nM) for 20 min at 37 °C in the dark. After washing with PBS, the cells were examined using the FITC channel on a flow cytometer. CCCP, a mitochondrial oxidative phosphorylation uncoupler, was used as the positive control.

### 2.13. Western Blotting

Proteins were transferred from the gel onto nitrocellulose or PVDF membranes that can bind proteins. The membranes were blocked with a solution to prevent nonspecific antibody binding.

### 2.14. Immunocytochemistry

Following the RF treatment, A549 cells were washed with PBS, fixed, and permeabilized with 2% paraformaldehyde (*w*/*v*) diluted in 0.5% Triton X-100 (*v*/*v*) for 30 min. The fixed cells were blocked with 5% BSA (*w*/*v*) in PBS containing 0.1% Tween 20 (PBST) for 1 h at room temperature. Subsequently, cells were incubated with anti-4-HNE in 1% BSA (*w*/*v*) in PBST overnight at 4 °C. After washing with 1% BSA (*w*/*v*) in PBST, the cells were incubated with Alexa Fluor-conjugated secondary antibodies (diluted 1:1000 in 1% BSA (*w*/*v*) in PBST) for 1 h at room temperature. Nuclei were stained with Hoechst 33342 (20 μg/mL) diluted in PBS for 15 min at room temperature. Finally, the cells were mounted in PLUS Antifade Mounting Medium (Vector Labs, Newark, CA, USA) and images were captured using a Nikon Ts2 fluorescence microscope (Tokyo, Japan).

### 2.15. Statistical Analysis

Data are presented as mean ± standard deviation (SD). Experiments were statistically analyzed using the Student’s *t*-test and differences were considered significant at *p* < 0.05, (*), *p* < 0.01 (**), and *p* < 0.001 (***).

## 3. Results

### 3.1. The Molecular Structures of RF and Its Cytotoxic Effects on Various Cancer Cell Lines

**Figure 1:**
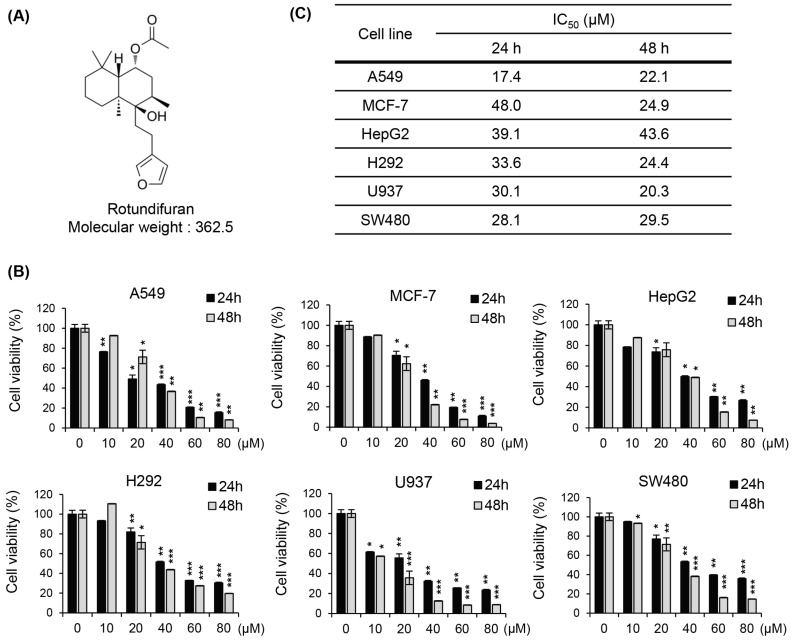
RF inhibited the growth of various cancer cell lines. (**A**) The molecular structure of RF. (**B**) Cell viability of A549, MCF-7, HepG2, H292, U937, and SW480 treated with RF at the indicated concentrations and time. (**C**) IC_50_ values of RF in various cell lines. All data are presented as the mean ± SD from a minimum of three independent experiments. Statistical differences were assessed using one-way ANOVA. * *p* < 0.05, ** *p* < 0.01, and *** *p* < 0.001 compared to the control. RF, rotundifuran.

RF is a natural compound with a bicyclic structure consisting of two fused rings (a furan ring and a cyclohexenone ring) and a lactone group. The molecular formula of RF is C_22_H_34_O_4_, with a molecular weight of 362.5 g/mol, and it is characterized by various functional groups, including a carbonyl group, an ether group, and a double bond. The unique molecular structure of RF is believed to be responsible for its diverse biological activities, including potential anti-cancer effects. A graphical representation of the molecular structure of RF is shown in Figure 1A.

In this study, we explored the effects of RF on the growth of diverse cancer cells. The human cancer cell lines used were A549 (lung cancer cells), MCF-7 (breast cancer cells), HepG2 (hepatocellular carcinoma cells), H292 (pulmonary mucoepidermoid carcinoma cells), U937 (human monoblastic leukemia cells), and SW480 (human colon cancer cells). The cells were treated with different concentrations of RF ranging from 0 to 80 µM, and the cell viability was assessed using the MTT assay. The MTT assay showed that RF inhibited the growth of all cancer cell lines in a dose-dependent manner. At lower concentrations of RF (up to 10 µM), a slight decrease in cell viability was observed, but the effect was not significant. However, at higher concentrations (20–80 µM), a significant decrease in cell viability was observed in all cancer cell lines (Figure 1B). The IC_50_ values of RF against each cell line are shown in Figure 1C.

### 3.2. Apoptosis Does Not Play a Critical Role in the Cell Death of Cancer Cells Induced by RF

**Figure 2:**
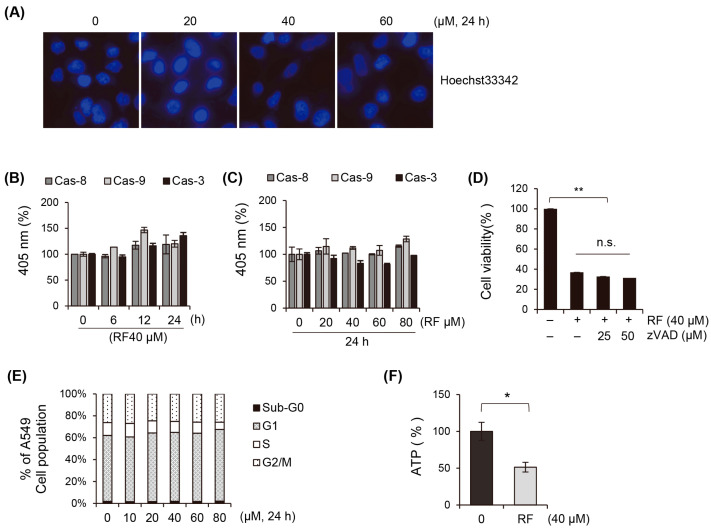
RF did not induce apoptosis. (**A**) Fluorescence microscopy images of A549 cells treated with RF at the indicated concentrations for 24 h and stained with Hoechst33342 stain to observe DNA cleavage. (**B**,**C**) Caspase 3, 8, and 9 activities of A549 cells treated with RF at different concentrations and incubation times. (**D**) Cell growth and viability of A549 cells pre-treated with pan-caspase inhibitor and RF for 24 h using the MTT assay. (**E**) Cell cycle analysis of A549 cells treated with the indicated concentrations of RF using flow cytometry after PI staining. (**F**) Intracellular ATP levels of the 24 h culture of A549 cells treated with 40 μM RF. All data are expressed as the mean ± SD obtained from at least three independent experiments. Statistical differences were analyzed using one-way ANOVA calculation. * *p* < 0.05 and ** *p* < 0.01 compared to the control. RF, rotundifuran; n.s., not significant.

We investigated the effects of RF on A549 cell death and explored the roles of apoptosis and necrosis in RF-induced cell death. Hoechst33342 staining confirmed that there were no segmental changes in the nucleus, which supported the fact that there was no apoptosis or DNA fragmentation caused by the RF treatment (Figure 2A). The activities of caspases 8, 9, and 3 were also examined because these caspases are executors of the apoptotic pathway. However, the RF treatment did not affect the activity of these caspases, suggesting that the apoptotic pathway is not involved in RF-induced cancer cell death (Figure 2B). In addition, the treatment with pan-caspase inhibitors, which universally block the activation of caspases, did not affect the cell growth inhibition induced by the RF treatment, further supporting the conclusion that apoptosis is not the primary mechanism of RF-induced cell death (Figure 2C,D). The cell cycle was also analyzed to investigate whether RF-induced cell death was associated with cell cycle arrest. However, our study did not observe any arrest in specific cell cycle phases or an increase in the sub-G1 cell group, indicating that RF-induced cell death is not associated with cell cycle arrest (Figure 2E). Instead, ATP depletion was observed after the RF treatment. ATP depletion is a hallmark of necrosis (Figure 2F). Given that apoptosis is not significantly involved in RF-induced cell death, we hypothesized that other mechanisms, such as necrosis or ferroptosis, may play a more substantial role in RF-induced cell death.

### 3.3. RF-Induced Autophagy Is Not Associated with Cell Death

**Figure 3:**
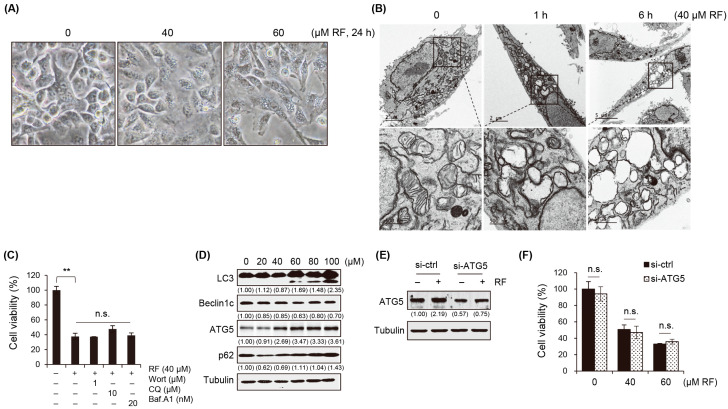
RF-induced autophagy was unrelated to cell death. (**A**) Morphological alterations in A549 cells observed under a microscope after exposure to 40 µM RF. (**B**) Transmission electron microscopy images of A549 cells treated with 40 µM RF for 1 or 6 h. (**C**) Cell viability of A549 cells pre-treated with the autophagy inhibitors wortmannin (Wort), chloroquine (CQ), and bafilomycin A1 (Baf.A1) were pre-treated for 1 h and then treated with 40 μM RF for 24 h using the MTT assay. (**D**) Western blot for autophagy-related genes in A549 cells treated with RF at the indicated concentrations for 24 h. (**E**) Western blot for ATG5 in non-targeted siRNA or ATG5 siRNA-mediated gene knockdown A549 cells in presence or absence of RF. (**F**) Cell viability of mock and ATG5 knockdown cells treated with RF at the indicated concentrations for 24 h using MTT. All data are expressed as the mean ± SD obtained from at least three independent experiments. Statistical differences were assessed using one-way ANOVA. ** *p* < 0.01 compared to the control. RF, rotundifuran; n.s., not significant.

Next, we investigated the effects of RF on autophagy. The results showed that the treatment with RF led to the formation of vacuoles in the cells, as confirmed with optical microscopy (Figure 3A). Electron microscopy showed that when treated with RF, mitochondria swelled, their cristae structure was destroyed, and they turned into vacuoles. These findings suggested that vacuole formation is a potential mechanism for RF-induced cell death (Figure 3B). To investigate whether the vacuoles formed were related to autophagy, the autophagy inhibitors wortmannin (Wort), chloroquine (CQ), and bafilomycin A1 (Baf.A1) were used. Treatments with these inhibitors did not alter the inhibitory effect on cell growth (Figure 3C) or vacuole formation. This suggested that vacuole formation in response to the RF treatment may not have been directly related to autophagy. Further analyses were performed to investigate the effects of RF on autophagy. The expression of autophagy marker proteins, such as LC3, Beclin1, ATG5, and p62, was examined. The results confirmed that treatment with more than 60 μM RF affected the expression of these proteins. The increase in LC3 cleavage, decrease in Beclin1 expression, reduction in ATG5 levels, and elevation of p62 levels indicated the induction of autophagy (Figure 3D, Supplementary Figure S1). To confirm the role of autophagy in RF-induced cell death, the effect of the ATG5 knockout (KO) was examined in ATG5^−/−^ MEF cells. The results showed that the cell growth inhibitory effect of RF was maintained even in the ATG5 KO cells (Figure 3E,F). This suggested that autophagy is not critical in RF-induced cell death. The formation of vacuoles in response to the RF treatment suggested that alternative mechanisms, such as necrosis and ferroptosis, may be involved in cell death.

### 3.4. RF Induces Ferroptotic Cell Death

**Figure 4:**
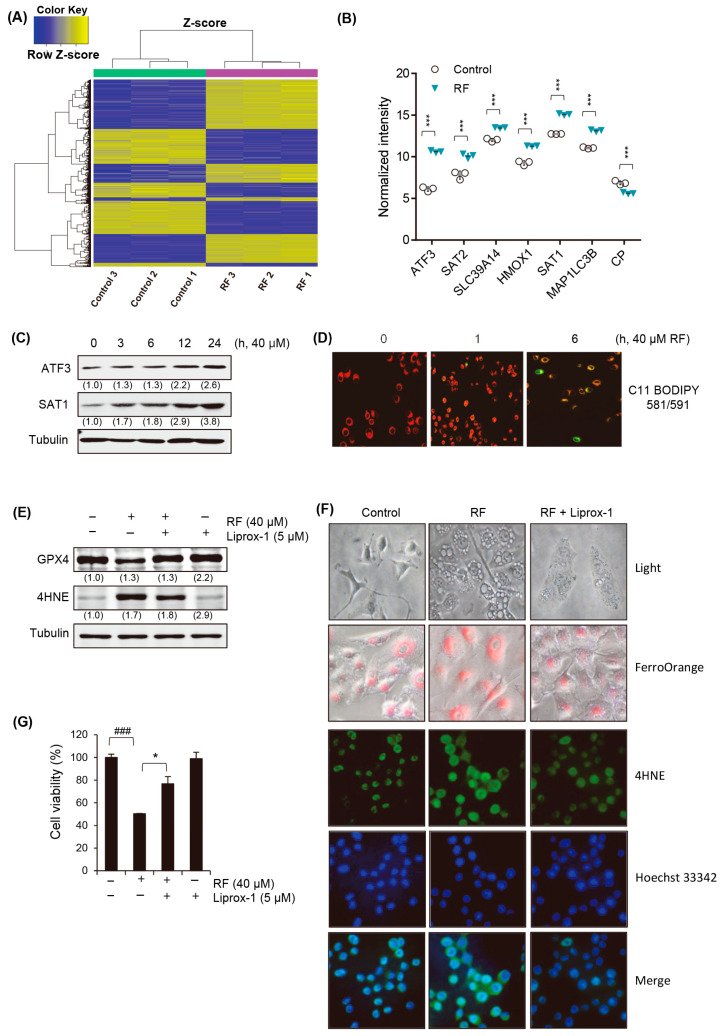
RF induced ferroptosis. (**A**,**B**) Microarray data from three independent experiments on A549 cells treated with RF. (**C**) Western blot for ATF3 and SAT1 in A549 cells exposed to 40 μM RF for the indicated time. (**D**) Lipid peroxidation in A549 cells exposed to 40 μM RF for the indicated time, examined using C11 BODIPY 581/591, with a shift in fluorescence from red to green upon oxidation. BODIPY intensity was quantified using a fluorescence microscope. (**E**–**G**) Cells were exposed to 40 μM RF for 24 h with a 1 h pre-treatment with 5 μM liproxstatin-1 (Liprox-1). (**E**) Western blotting for ATF3 and SAT1 expression. (**F**) Vacuole formation and intracellular ferrous visualized using FerroOrange staining (upper panel), while immunocytochemistry was performed to visualize 4HNE expression (bottom panel) under fluorescence microscopy. (**G**) Cell viability was analyzed with MTT. All data are expressed as the mean ± SD obtained from at least three independent experiments. Statistical differences were assessed using one-way ANOVA. * *p* < 0.05 and *** *p* < 0.001 compared to the RF-treated group. ^###^ *p* < 0.001 compared to the control. RF, rotundifuran.

The mechanism underlying RF-induced cell death was investigated using a cDNA chip analysis to detect changes in gene expression. The results showed that the RF treatment dramatically induced significant changes in gene expression (Figure 4A). Among these, only the ferroptosis-related genes were selected, as shown in Figure 4B. The significant upregulation of ferroptosis-related genes, including ATF3 (activating transcription factor 3), SAT2 (spermidine/Spermine N1-acetyltransferase 2), SLC39A14 (solute carrier family 39 member 14), HMOX1 (heme oxygenase 1), SAT1 (spermidine/spermine N1-acetyltransferase 1), and MAP1LC3B (microtubule-associated protein 1 light chain 3 beta), was observed in response to the RF treatment. The log_2_-fold changes in gene expression for each of these genes were as follows: ATF3, 22.4-fold upregulation; SAT2, 4.9-fold upregulation; SLC39A14, 2.7-fold upregulation; HMOX1, 4.1-fold upregulation; SAT1, 5.1-fold upregulation; and MAP1LC3B, 2.3-fold upregulation.

The Western blot analysis confirmed the protein-level changes in ATF3 and SAT2, which exhibited the most significant increase in mRNA expression in response to the RF treatment. Notably, both proteins showed a time-dependent increase in levels, reaching their peak at 24 h (Figure 4C, Supplementary Figure S2). Data obtained using C11 BODIPY 581/591 to detect lipid peroxidation during ferroptosis demonstrated a time-dependent change in fluorescence from red to green (Figure 4D). This shift in fluorescence was indicative of an alteration in the oxidation state of the C11 BODIPY probe and provided valuable insights into the kinetics of lipid peroxidation during the ferroptotic process. To further explore the role of ferroptosis in RF-induced cell death, we pre-treated the cells with a ferroptosis inhibitor (liproxstatin-1) and examined cell growth inhibition and vacuole formation. The treatment with RF induced the typical features of ferroptosis, including GPX4 depletion and 4HNE accumulation (Figure 4E). Additionally, vacuole formation, the intracellular ferrous ion concentration, and 4HNE accumulation induced by RF were inhibited by the treatment with liproxstatin-1 (Figure 4F). Ultimately, the treatment with liproxstatin-1 significantly increased the cell death induced by RF (Figure 4G), suggesting that ferroptosis may be involved in cell death and vacuole formation induced by RF.

### 3.5. Cytosolic Calcium Concentration Is Elevated upon RF-Induced Ferroptosis

**Figure 5:**
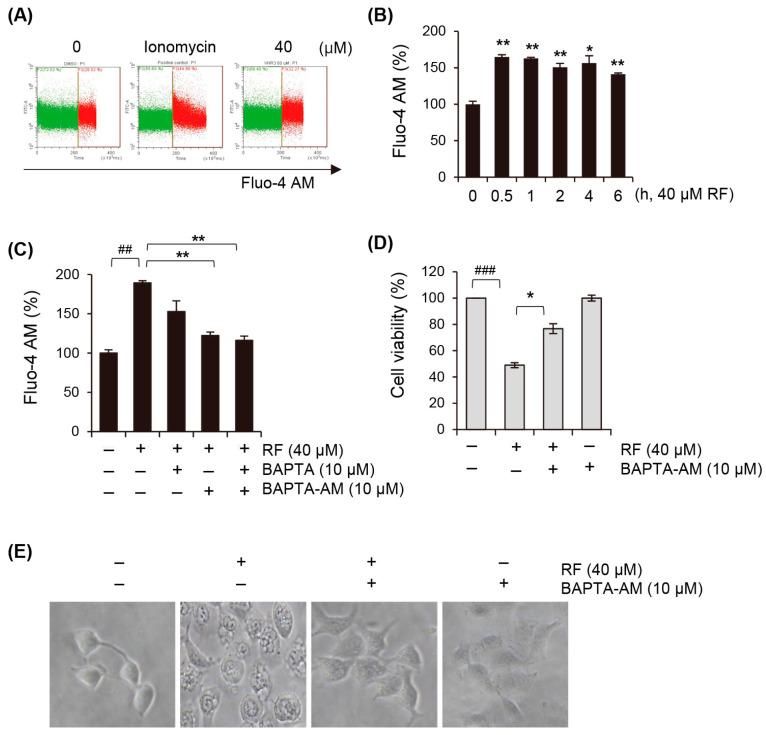
RF increased cytosolic calcium concentration. (**A**) Flow cytometry analysis of A549 cells treated with 20 and 40 µM RF for 30 min and stained with 2 μg/mL Fluo-4 AM. (**B**) Changes in cytoplasmic calcium levels in A549 cells exposed to 40 μM RF at the indicated times, assessed through staining with Fluo-4 AM. (**C**–**E**) A549 cells were pre-treated with 10 µM BAPTA, 10 µM BAPTA-AM, or 10 µM BAPTA plus 10 µM BAPTA-AM after exposure to 40 µM RF. (**C**) Changes in intracellular calcium concentration analyzed with Fluo-4 AM. Cell viability evaluated using MTT. (**E**) Vacuole formation observed using an optical microscope. Statistical differences were assessed using one-way ANOVA. * *p* < 0.05 and ** *p* < 0.01 compared to the RF-treated group. ^##^ *p* < 0.01 and ^###^ *p* < 0.001 compared to the control. RF, rotundifuran.

Recent studies have shown that calcium Ca^2+^ signaling plays a crucial role in ferroptosis [26,27]. Next, we investigated the relationship between calcium levels and ferroptosis in RF-treated cells. Fluo-4 AM-pre-stained A549 cells were exposed to RF and intracellular calcium changes were monitored on a second-by-second basis. The results revealed that the positive control ionomycin led to a rapid increase in calcium levels within seconds of exposure, followed by a return to basal levels. In contrast, RF exhibited a sustained increase in calcium levels within a short timeframe (Figure 5A). To further investigate the changes in calcium concentration induced by the RF treatment, we examined variations over time. The experimental findings indicated that at 0.5 h, the calcium levels reached their highest concentration and gradually decreased thereafter until 6 h (Figure 5B). This suggested that calcium signaling is involved in the induction of ferroptosis by RF. Furthermore, to confirm the role of calcium in RF-induced cell death, cells were pre-treated with the calcium inhibitors BAPTA and BAPTA-AM, which inhibited the calcium increase induced by RF (Figure 5C). Notably, this pre-treatment significantly reversed the inhibition of cell growth (Figure 5D) and vacuole formation (Figure 5E) induced by RF, providing further evidence that calcium plays a critical role in RF-induced ferroptosis and cell death.

### 3.6. RF Induces Mitochondrial Dysfunction by Excess Calcium

**Figure 6:**
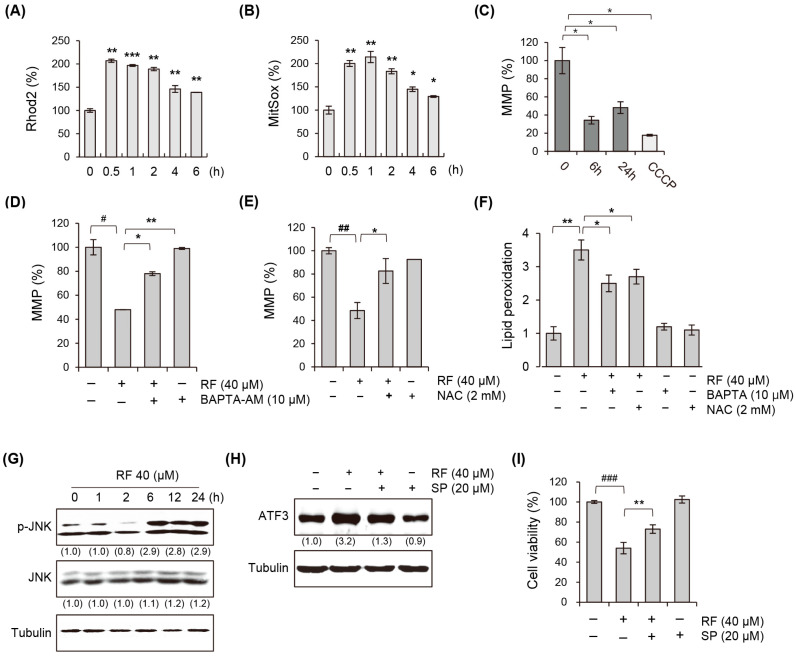
RF induced changes in the mitochondrial dynamics of A549 cells. (**A**) Mitochondrial calcium levels in A549 cells treated with 40 µM RF for each indicated time period, visualized using Rhod2 staining. (**B**) Mitochondrial superoxide levels in A549 cells treated with 40 µM RF for each indicated time period, assessed using MitoSox staining. (**C**) The MMP of A549 cells treated with 40 µM RF. CCCP was used as a positive control. (**D**) Changes in MMP after pre-treatment with 10 µM BAPTA-AM and treatment with 40 µM RF for 24 h. (**E**) Changes in MMP after pre-treatment with 2 mM NAC and treatment with 40 µM RF for 24 h. (**F**) Lipid peroxidation levels after pre-treatment with 10 µM BAPTA, 2 mM NAC, and 40 µM RF for 24 h. (**G**) Western blot for p-JNK and JNK in A549 cells exposed to 40 μM of RF at the indicated times. (**H**) Western blotting for ATF3 in A549 cells pre-treated with 20 μM SP600125, a JNK inhibitor, and treatment with 40 μM RF for 24 h. (**I**) MTT analysis of A549 cells pre-treated with 20 μM SP600125, a JNK inhibitor, and treatment with 40 μM RF for 24 h. Statistical differences were assessed using one-way ANOVA. * *p* < 0.05, ** *p* < 0.01, and *** *p* < 0.001 compared to the RF-treated group. ^#^ *p* < 0.05, ^##^ *p* < 0.01 and ^###^ *p* < 0.001 compared to the control. RF, rotundifuran.

We measured mitochondrial calcium and ROS levels to investigate the role of mitochondrial dynamics in RF-induced ferroptosis. Our results showed that treatment with RF led to an increase in the concentration of calcium in the mitochondria, which, in turn, increased the production of ROS (Figure 6A,B). The increase in mitochondrial calcium and ROS production was accompanied by a reduction in the mitochondrial membrane potential (MMP), which is a characteristic of ferroptosis (Figure 6C). To investigate the role of excess calcium and ROS production in the decrease in MMP induced by RF, we pre-treated cells with the calcium chelator BAPTA-AM and the ROS scavenger NAC. We found that the pre-treatment with these compounds significantly restored the MMP upon the RF treatment (Figure 6D,E). Additionally, these inhibitors significantly reduced lipid peroxidation (Figure 6F), suggesting that the regulation of calcium and ROS homeostasis is critical for maintaining MMP and preventing ferroptosis.

JNK is a stress-activated protein kinase activated by various stimuli, including oxidative stress and calcium signaling. Our results showed that the RF treatment induced JNK phosphorylation in a time-dependent manner (Figure 6G, Supplementary Figure S3). Additionally, pre-treatment with the JNK inhibitor SP600125 suppressed the expression of ATF3, a protein associated with RF-induced ferroptosis (Figure 6H, Supplementary Figure S3). This inhibition of JNK signaling and subsequent reduction in ATF3 expression led to the alleviation of ferroptosis-dependent cell growth inhibition (Figure 6I). These findings suggested that the JNK signaling pathway may play a crucial role in RF-induced ferroptosis and cell death in A549 cells and that JNK may be a crucial regulatory molecule for RF-mediated ferroptosis.

## 4. Discussion

Lung cancer is a disease with a high mortality rate and is a major public health concern. Ferroptosis is a recently identified form of regulated cell death characterized by the accumulation of lipid peroxides. Recent research suggests a potential association between ferroptosis and cancer formation and suppression [28,29]. Ferroptosis inhibits tumorigenesis in lung cancer, and understanding the mechanisms underlying ferroptosis in lung cancer is crucial for developing new strategies for its prevention and treatment [29]. RF, a compound used in Chinese medicine, is obtained from the plant species *V. rotundifolia*. In the current study, the treatment of various cancer cells with RF inhibited cell growth and induced cell death, which was characterized by the formation of vacuoles derived from the mitochondria.

The cDNA chip analysis revealed that the expression levels of several ferroptosis-related genes were altered in RF-treated cells. These genes included ATF3 (activating transcription factor 3), SAT2 (spermidine/spermine N1-acetyltransferase 2), SLC39A14 (solute carrier family 39 member 14), HMOX1 (heme oxygenase 1), and SAT1 (spermidine/spermine N1-acetyltransferase 1). These genes play important roles in various cellular processes, including lipid metabolism, iron homeostasis, oxidative stress, and inflammation, all of which are closely related to the pathogenesis of cancer. The upregulation of these genes suggested that ferroptosis may be involved in RF-induced cell death. ATF3 is a stress-responsive transcription factor that regulates cellular responses to oxidative stress. Its upregulation may indicate a protective mechanism triggered in response to ferroptotic stress as it can modulate the expression of genes involved in lipid peroxidation and iron metabolism, potentially mitigating ferroptosis. SAT1 and SAT2 play roles in polyamine metabolism and influence cellular sensitivity to oxidative stress. The upregulation of SAT2 suggested a response to counteract the oxidative stress associated with ferroptosis, potentially leading to increased polyamine levels for cell protection. Furthermore, SLC39A14 regulates intracellular zinc levels, which can affect ferroptosis by modulating iron metabolism and oxidative stress. Its upregulation may reflect an attempt to modulate these processes in response to ferroptotic stress, potentially as a cellular defense mechanism. HMOX1 degrades heme and releases iron. The upregulation of HMOX1 may indicate a cellular response to heme-induced oxidative stress with the aim of managing iron levels and mitigating ferroptosis. MAP1LC3B is a key player in autophagy, a process that influences ferroptosis by eliminating damaged cellular components, including mitochondria. The upregulation of MAP1LC3B may indicate the activation of autophagic pathways in response to ferroptotic stress, potentially contributing to cellular protection. The identification of these genes provided important insights into the mechanisms of ferroptosis and potential targets for developing new cancer therapies. In addition, the cDNA chip analysis showed that the expression of a group of ER stress-related genes was significantly increased by the RF treatment (Supplementary Figure S4 and Table S1). Further studies are needed to confirm the role of ferroptosis in RF-induced cell death and identify specific ferroptosis-related targets for the development of new therapies.

In the field of cancer therapy, autophagy is categorized into three principal types: (i) cytoprotective autophagy, which enables cancer cells to withstand stress by preserving cellular balance, thus enhancing their resistance to therapeutic interventions; (ii) cytotoxic autophagy, which leads to cellular demise, serving as a pathway through which cancer therapies manifest their effectiveness; and (iii) nonprotective autophagy, characterized by its indifferent effect on a tumor cell’s reaction to external stressors, such as chemotherapy or radiation, with its suppression showing no significant influence on treatment results, as evidenced by research involving RF. Our investigations into RF have demonstrated its capacity to trigger autophagy in cancer cell lines, as shown by LC3 cleavage and diminished ATG5 levels. RF also induced the formation of intracellular vesicles and curtailed the proliferation of cancer cells. However, inhibiting autophagy prior to RF exposure did not negate the effects on vesicle formation or the reduction in cell growth caused by RF, suggesting that RF induces a form of autophagy that is nonprotective.

Calcium has been shown to be involved in ferroptosis, a form of regulated cell death characterized by the accumulation of lipid peroxides. Previous reports indicated that calcium signaling can modulate ferroptosis through various mechanisms. One way that calcium can modulate ferroptosis is by regulating the iron metabolism. Calcium can regulate the expression and activity of proteins involved in iron transport, storage, and metabolism, which, in turn, can affect intracellular iron levels and the susceptibility of cells to ferroptosis. Additionally, calcium regulates the activity of enzymes involved in lipid peroxidation, a key feature of ferroptosis. For example, calcium can activate phospholipase A2 (PLA2), which can promote the release of fatty acids from phospholipids, and lipoxygenases (LOX), which can catalyze the oxidation of polyunsaturated fatty acids and the production of lipid peroxides. Calcium also regulates the activity of glutathione peroxidase 4 (GPX4), a key enzyme involved in lipid peroxide detoxification. Calcium signaling can affect GPX4 activity by regulating the availability of glutathione, a key cofactor of GPX4, or by modulating the expression and activity of selenoproteins, a group of proteins that include GPX4 and require selenium for their activity. Overall, calcium plays a complex and multifaceted role in ferroptosis. Further research is needed to fully elucidate the underlying relationship between calcium signaling and ferroptosis.

When the concentration of calcium in the cytosol increases, calcium ions can move into the mitochondria through specific transporters, such as the mitochondrial calcium uniporter (MCU) complex. The MCU complex is a transmembrane protein that spans the inner mitochondrial membrane and allows calcium ions to enter the mitochondrial matrix. Once inside the matrix, calcium can regulate various mitochondrial processes, including oxidative phosphorylation and the maintenance of MMP. However, excessive calcium uptake into the mitochondria can lead to mitochondrial dysfunction and cell death, including ferroptosis-mediated cell death [30,31]. We used Rhod2, a fluorescent probe for mitochondrial calcium imaging, to investigate the movement of cytoplasmic calcium to the mitochondria in cells treated with RF. Our study revealed a time-dependent increase in mitochondrial calcium levels in response to the RF treatment (Figure 6A). Excess calcium within mitochondria has been shown to increase ROS production. This phenomenon can be attributed to the dysfunction of the electron transport chain, which occurs because of high levels of calcium. Additionally, calcium overload can activate enzymes responsible for producing ROS, including nitric oxide synthase and NADPH oxidase [32,33]. Further, we measured the amount of superoxide in the mitochondria after treatment with RF using MitoSox, a fluorescent probe for superoxide detection. Our results showed a time-dependent increase in MitoSOX fluorescence, indicating the accumulation of superoxide in the mitochondria following the RF treatment. This suggested that the excess mitochondrial calcium induced by RF may lead to the generation of ROS and contribute to the cell death observed in ferroptosis. Excess calcium and ROS production in mitochondria can decrease MMP [34,35]. Calcium overload can cause electron transport chain dysfunction, leading to decreased MMP. Additionally, ROS accumulation can damage the mitochondrial membrane, resulting in decreased MMP. The disruption of MMP can have significant consequences on cellular processes, including ATP synthesis and apoptosis, and has been implicated in various diseases. Furthermore, MMP disruption is a hallmark of ferroptosis and plays a critical role in its initiation and progression [36,37]. It has been shown that the decrease in MMP can lead to the loss of mitochondrial function and the release of iron into the cytoplasm, which can catalyze lipid peroxidation and trigger ferroptosis. Therefore, the regulation of MMP is crucial for maintaining cellular homeostasis and preventing cell death via ferroptosis.

JNK is a stress-activated protein kinase activated by various stimuli, including oxidative stress and calcium signaling. Upon activation, JNK phosphorylates and regulates the expression and activity of various downstream effectors, including transcription factors, kinases, and phosphatases. During ferroptosis, JNK signaling plays a crucial role in regulating the expression and activity of various enzymes involved in the lipid metabolism, including ATF3, ACSL4 (long-chain acyl-CoA synthetase 4), and GPX4 (glutathione peroxidase 4). The activation of JNK promotes the downregulation of GPX4, a key enzyme that can prevent lipid peroxidation and ferroptosis. Additionally, JNK upregulates ACSL4, which promotes the accumulation of lipid peroxides and contributes to ferroptosis. These results indicated that JNK activation is necessary for RF-induced ATF3. JNK signaling is an important pathway that links ROS and lipid metabolism to ferroptosis and may be a potential therapeutic target for the treatment of ferroptosis-related diseases.

Taken together, our results showed, for the first time, that RF inhibits cell growth by inducing ferroptosis in a lung cancer cell line. Our findings revealed that the induction of ferroptosis by RF was associated with calcium signaling, ROS generation, and JNK signaling.

## Data Availability

All data analyzed in this study are available from the corresponding author (mokim@kribb.re.kr) upon request.

## Supplementary Materials

The following supporting information can be downloaded at: https://www.mdpi.com/article/10.3390/biomedicines12030576/s1, Figure S1: Original Western blots in Figure 3; Figure S2: Original Western blots in Figure 4; Figure S3: Original Western blots in Figure 6; Figure S4: Changes in ER stress-related genes induced by the RF treatment based on the microarray results; Table S1: Changes in ER stress-related gene expression after the RF treatment in A549 cells.
