# Supplementary material for: Rotundifuran Induces Ferroptotic Cell Death and Mitochondria Permeability Transition in Lung Cancer Cells
Source: Biomedicines. 2024 Mar 5;12(3):576. doi: 10.3390/biomedicines12030576 (PMC10968504; doi:10.3390/biomedicines12030576)
Supplement: Supplementary file 1 [file biomedicines-12-00576-s001.zip › biomedicines-2860521-supplementary.pdf]

Supplementary Information

Rotundifuran Induces Ferroptotic Cell Death and Mitochondria Permeability Transition in Lung Cancer Cells

Myung-Ji Kang *et al.*

● Supplementary Figures

Figure S1. Original western blots in Figure 3 2

Figure S2. Original western blots in Figure 4 3

Figure S3. Original western blots in Figure 6 4

Figure S4. The changes in ER stress-related genes induced by RF treatment based on the microarray results 5

● Supplementary Table

Table S1. The list of ER stress-related genes changed by RF treatment in A549 cells 6

(A)

Target	Size	Original image
LC3	14, 16 kDa	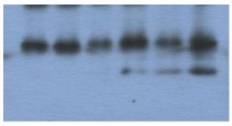
Beclin1c	52 kDa	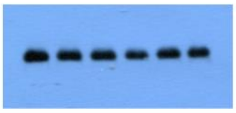
ATG5	32 kDa	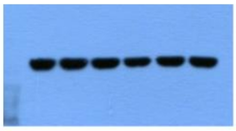
p62	62 kDa	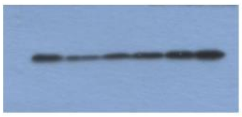
Tubulin	50 kDa	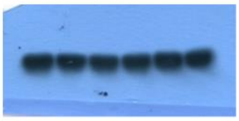

(B)

Target	Size	Original image
LC3	14, 16 kDa	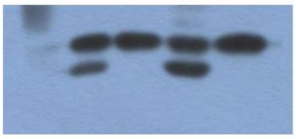

Figure S1. Original western blots in Figure 7. **(A)** Uncropped and unprocessed images of Figure 3D. **(B)** Original images of Figure 3E.

(A)

Target	Size	Original image
SAT1	25 kDa	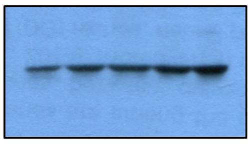
ATF3	21 kDa	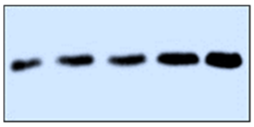
Tubulin	50 kDa	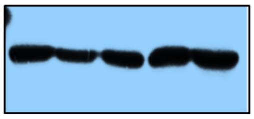

(B)

Target	Size	Original image
GPX4	20 kDa	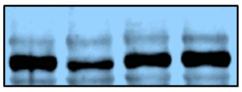
4HNE	67 kDa	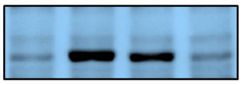
Tubulin	50 kDa	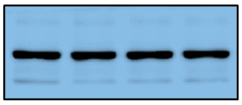

Figure S2. Original western blots in Figure 4. **(A)** Uncropped and unprocessed images of Figure 4C. **(B)** Original images of Figure 4E.

(A)

Target	Size	Original image
p-JNK	46, 54 kDa	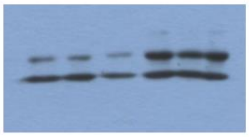
JNK	46, 54 kDa	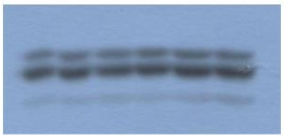
Tubulin	50 kDa	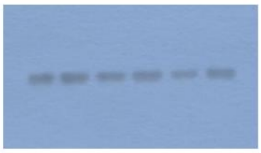

(B)

Target	Size	Original image
ATF3	21 kDa	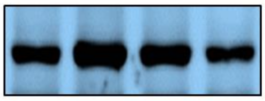
Tubulin	50 kDa	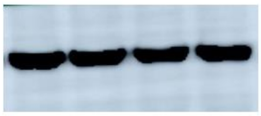

Figure S3. Original western blots in Figure 6. **(A)** Uncropped and unprocessed images of Figure 6G. **(B)** Original images of Figure 6H.

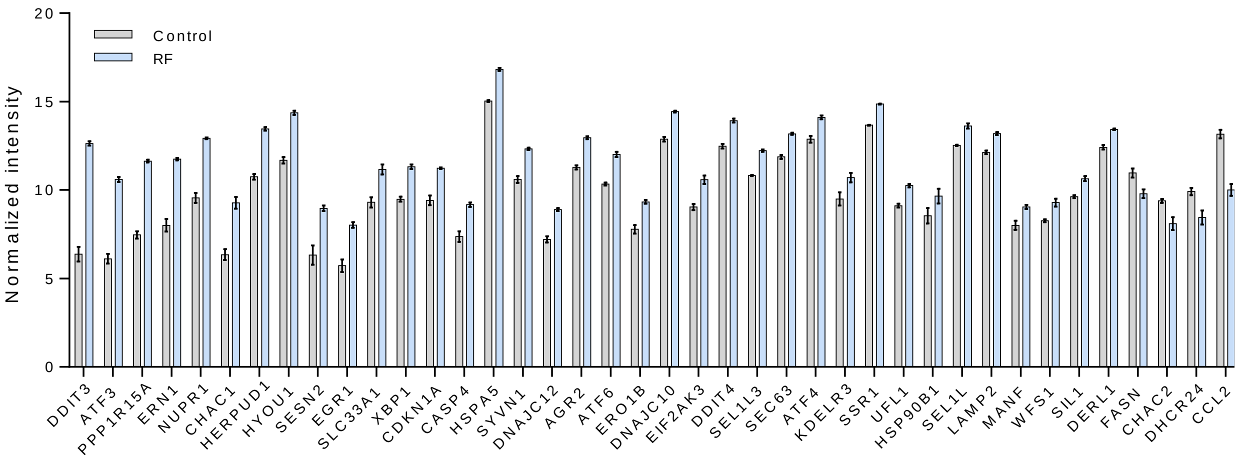

Figure S4. The changes in ER stress-related genes induced by RF treatment based on the microarray results. Microarray data were obtained from three independent experiments in A549 cells treated with RF. The data were represented as a bar graph, illustrating the normalized intensity.

Table S1. The list of ER stress-related genes changed by RF treatment in A549 cells

Gene	Full name	Log2 fold change	Gene	Full name	Log2 fold change
DDIT3	DNA-damage-inducible transcript 3	76.6	DNAJC10	DnaJ (Hsp40) homolog, subfamily C, member 10	2.9
ATF3	activating transcription factor 3	22.4	EIF2AK3	eukaryotic translation initiation factor 2-alpha kinase 3	2.9
PPP1R15A	protein phosphatase 1, regulatory subunit 15A	18.0	DDIT4	DNA damage inducible transcript 4	2.7
ERN1	endoplasmic reticulum to nucleus signaling 1	13.3	SEL1L3	sel-1 suppressor of lin-12-like 3 (C. elegans)	2.7
NUPR1	nuclear protein 1, transcriptional regulator	10.4	SEC63	SEC63 homolog, protein translocation regulator	2.5
CHAC1	ChaC glutathione-specific gamma-glutamylcyclotransferase 1	7.6	ATF4	activating transcription factor 4	2.4
HERPUD1	homocysteine-inducible, endoplasmic reticulum stress-inducible, ubiquitin-like domain member 1	6.5	KDELR3	KDEL (Lys-Asp-Glu-Leu) endoplasmic reticulum protein retention receptor 3	2.3
HYOU1	hypoxia up-regulated 1	6.5	SSR1	signal sequence receptor, alpha	2.3
SESN2	sestrin 2	6.2	UFL1	UFM1-specific ligase 1	2.2
EGR1	early growth response 1	4.9	HSP90B1	Transcript Identified by AceView, Entrez Gene ID(s) 7184	2.2
SLC33A1	solute carrier family 33 (acetyl-CoA transporter), member 1	3.6	SEL1L	sel-1 suppressor of lin-12-like (C. elegans)	2.1
XBP1	X-box binding protein 1	3.6	LAMP2	lysosomal-associated membrane protein 2	2.1
CDKN1A	cyclin-dependent kinase inhibitor 1A (p21, Cip1)	3.5	MANF	mesencephalic astrocyte-derived neurotrophic factor	2.1
CASP4	caspase 4	3.5	WFS1	Wolfram syndrome 1 (wolframin)	2.0
HSPA5	heat shock 70kDa protein 5 (glucose-regulated protein, 78kDa)	3.4	SIL1	SIL1 nucleotide exchange factor	2.0
SYVN1	synovial apoptosis inhibitor 1, synoviolin	3.3	DERL1	derlin 1	2.0
DNAJC12	DnaJ (Hsp40) homolog, subfamily C, member 12	3.2	FASN	fatty acid synthase	-2.3
AGR2	anterior gradient 2, protein disulphide isomerase family member	3.2	CHAC2	ChaC, cation transport regulator homolog 2 (E. coli)	-2.4
ATF6	activating transcription factor 6	3.2	DHCR24	24-dehydrocholesterol reductase	-2.8
ERO1B	endoplasmic reticulum oxidoreductase beta	2.9	CCL2	chemokine (C-C motif) ligand 2	-8.9
